# Health-Related Quality of Life and Work Satisfaction in Working-Aged Adults Pre- and Post-Cochlear Implant: A Longitudinal Study

**DOI:** 10.3390/jcm11237024

**Published:** 2022-11-28

**Authors:** Maja Magdalena Olsson, Aaran Thomas Lewis, Louise Arvidsson, Håkan Hua

**Affiliations:** 1 Department of Public Health, School of Health Sciences, University of Skövde, 541 28 Skövde, Sweden; 2 Centre for Healthcare Transformation, School of Nursing, Queensland University of Technology, Brisbane, QLD 4059, Australia; 3 Cochlear Ltd., 435 33 Mölnlycke, Sweden

**Keywords:** HRQoL, work satisfaction, hearing loss, cochlear implant, HUI-3, SSQ

## Abstract

Hearing loss is a growing public health concern associated with decreased health-related quality of life (HRQoL) and a negative impact on work life. Knowledge about the long-term benefits for patients receiving cochlear implants may provide knowledge imperative for policymakers to promote better HRQoL and working life outcomes for individuals with hearing loss. The purpose of this study was to explore how HRQoL, hearing disabilities, and work satisfaction outcomes changed in working-aged adults with severe to profound hearing loss from pre- to post-receiving a cochlear implant (CI) between the baseline, year one, and year two. This longitudinal study used Cochlear’s Implant Recipient Observational Study (IROS) registry data to assess HRQoL, hearing disabilities, and work satisfaction in 18–65-year-old CI recipients. Data were collected pre- and post-implantation at baseline, year one, and year two follow-up. One hundred and twenty-seven CI recipients participated in the study. Significant improvements were observed for HRQoL outcomes for hearing, speech, emotion, and health utility post-implant. Overall hearing disability decreased post-CI, and work satisfaction improved. With the increasing prevalence of hearing disabilities, this is pertinent knowledge that supports the use of CIs for hearing rehabilitation which may promote better HRQoL and work satisfaction.

## 1. Introduction

In 2021, the World Health Organization (WHO) launched a report that addressed hearing health as a global public health priority [1]. Hearing loss (HL) and deafness are rapidly growing health concerns estimated to affect 1.57 billion globally, which means that approximately one in five people suffers from hearing disabilities [2]. Hearing loss is associated with significant humanistic burdens, such as social isolation, loneliness, stigma, and reduced health-related quality of life (HRQoL) [1,3]. Hearing loss can be a major disability that imposes activity limitations and participation restrictions on social, physical, biological, medical/clinical, psychosocial, and normative levels [1,4]. Hence, HRQoL, i.e., the self-perceived functions of health status in an individual’s daily life activities [5], is often reduced in individuals with HL [1,3]. The condition also negatively affects work life, and worse hearing is related to decreased work performance and less social participation in the workplace [6] as well as a lower physical HRQoL [7]. Other aspects that make work life challenging for those with HL are communication difficulties, such as group interactions, disturbance by loud noises, etc. [8]. Individuals with HL may compensate for their disability in the workplace [9] by, for example, frequently asking colleagues to repeat what they said or by refraining from taking part in situations where their hearing impairment may be a problem [8]. There may also be social and mental health impacts of having HL in the workplace which include isolation, embarrassment, perceived insecurity, and inadequacy [7,10]. Economic burdens of HL also impact societies and individuals greatly, and estimations have shown that unaddressed hearing loss costs the global economy 980 billion USD annually [1].

To increase hearing function in individuals with HL, hearing technologies, such as hearing aids and cochlear implants (CIs), can be used to generate a representation of sounds from the environment [1,11]. CIs are hearing devices that bypass the middle- and inner-ear structures and stimulate the cochlea directly, providing access to sound and speech. The system consists of an implantable receiver/stimulator module with an electrode array, a microphone, and a sound processor. The external microphone collects sounds and sends them to the externally worn sound processor, which converts them into a digital signal. The signal is sent across intact skin to the implanted receiver coil and transmitted as an electrical current via an electrode that is threaded inside the cochlea. These electrodes stimulate the auditory nerve, which transmits the impulses along the auditory pathway. Previous studies have reported that CIs can increase HRQoL in adults with severe to profound HL [12,13,14] and may improve employment status, employment satisfaction, career development, and work opportunities [15]. Individuals with CIs have also shown positive outcomes in work-related performance, e.g., overall increased functioning, fewer concerns about being marginalised in the workplace, and a decreased fear of losing their job after implantation [15,16].

With a global growing prevalence of HL, it is pertinent to explore the long-term benefits for patients receiving CIs which may provide knowledge imperative for policymakers to promote better HRQoL and working life outcomes for individuals with HL. The overarching purpose of this study was, therefore, to explore how HRQoL, hearing disabilities, and work satisfaction outcomes changed in working-aged adults with severe to profound hearing loss from pre- to post-receiving CIs between baseline, year one follow-up (YR1), and year two follow-up (YR2).

## 2. Materials and Methods

This study had a longitudinal design with repeated measures at baseline, YR1, and YR2 follow-up. Participants in the study received three different questionnaires including measurements for generic HRQoL, levels of hearing disabilities, and work satisfaction at the three measure points. The study process and reporting of findings were guided by the checklist Strengthening the Reporting of Observational Studies in Epidemiology (STROBE) [17].

### 2.1. Study Population and Data Collection

Data was obtained from Cochlear’s international registry, the *Implant Recipient Observational Study* (IROS) [ClinicalTrials.gov NCT02004353] which contains data collected from clinicians and CI recipients between years 2009–2020 [18]. Individuals in the IROS registry were recruited from hearing health clinics in different countries by their clinicians. Recipients of CIs self-reported perceived hearing disabilities, HRQoL, and work satisfaction via online questionnaires in their own language. For the purpose of this study, all individuals with severe to profound HL of working age, i.e., between 18–65 years of age with one or several follow-ups were extracted from the IROS registry.

### 2.2. Ethical Considerations

The IROS study was approved by Ethical Review Boards of participating centres in Colombia (Clínica Rivas, CEL 5277), Germany (Medizinische Hochschule Hannover, 1241–2011), Hungary (Egészségügyi Nyilvántartási és Képzési Központ, 070662/2015/OTIG), Poland (*Uniwersytetu Medycznego w Łodzi,* RNN/117/12/KE), Spain (Hospital de la Santa Creu i Sant Pau, HSCSP 11/083), and South Africa (Stellenbosch University, N15/02/015) according to institutional and national research standards. Participants received verbal and written information about the purpose of the study before providing signed informed consent to participate.

### 2.3. Outcome Measures

Generic HRQoL was measured by Health Utilities Index Mark 3 (HUI-3), a 15 item survey with eight health attributes: vision, hearing, speech, ambulation, dexterity, emotion, cognition, and pain/complaints [19]. The instrument generates attribute scores and a health utility value which ranges from −0.36 (health state worse than death), 0.00 (death), to 1.00 (full/perfect health) [19]. Differences of 0.03 for the health utility value are considered clinically important, and differences of 0.01 may be contextually meaningful [19]. For the single attribute scores, differences of 0.05 are considered clinically important [19]. The validity and responsiveness of HUI-3 for individuals with HL have been reported as good as opposed to other HRQoL instruments, such as EQ-5D and SF-6D [20].

Hearing disabilities were measured by the 49 item instrument The Speech, Spatial and Qualities of Hearing Scale (SSQ), which measures hearing disabilities on a scale ranging from 0 (minimum/not at all, etc.) to 10 (maximum/ perfectly, etc.) across the domains of speech hearing, spatial hearing, and qualities of hearing [21]. Speech hearing encompasses perceptions of speech hearing in different contexts and how this may change in relation to the impact of, e.g., competing sounds and background conditions (quiet, noises, many voices at the same time, etc.) [21]. Spatial hearing assesses directional and distance judgements of sound in everyday situations, e.g., loudness because of spatial dynamics, which could be vocal emphasis or changes in emotional tone [21]. Qualities of hearing address whether sounds are recognisable and how clear and naturally they occur as well as the ability to experience simultaneous sounds as separated [21].

Employment status and work satisfaction were assessed by questions, e.g., if the participant was employed (yes/no), grade of employment (part-time/full-time), job classification (multiple choice categories), job title (free text), satisfaction with the job (yes/no), and if their hearing ability had negatively affected the daily work satisfactory (not at all/sometimes/most of the time/always). No definition was given for the concept of “satisfaction” as individuals assign different meanings to what they find satisfactory, and, as such, it was understood from the viewpoint of the participant’s personal interpretation. Unemployed participants were asked if they were retired (yes/no), their level of satisfaction being unemployed (yes/no/don’t know), and if they believed that their hearing ability was the reason for not being employed (yes/no/do not know). Employed participants were asked about the impact on hearing ability through the hearing device(s) related to the daily work, if there had been any changes over the last 12 months compared to before having the implant surgery (yes/no), and if the device(s) enabled them to do their job now (much better/marginally better/the same/marginally worse/much worse).

### 2.4. Data Analysis

Descriptive statistics were used for background information. Categorical variables are presented with number (*n*) and percentage. Continuous data, i.e., age, was calculated as a mean value with standard deviation (SD). The HUI-3 and SSQ subscale and global score data were analysed by Kolmogorov–Smirnov and Shapiro–Wilk tests and by examining histograms to determine the normality distribution. For the SSQ data median values were imputed for individuals who had occasionally missing data (<1%). Subscales and global score means for HUI-3 and SSQ as well as work satisfaction summary scores were compared between baseline and YR1 with Wilcoxon Signed rank test and with Friedman’s test between baseline and across YR1 and YR2. Categorical data were compared between baseline and YR1 with McNemar’s test and with Cochran’s Q test between baseline across YR1 and YR2. Statistical analyses were performed using the Statistical Package for the Social Sciences (SPSS) version 28.0. All statistical tests were conducted by using a significance level of *p* < 0.05.

## 3. Results

### 3.1. Study Sample and Subgroup Presentation

One hundred and twenty-seven IROS participants met the inclusion criteria and were included in the study. These had data for HUI-3 and SSQ at baseline, YR1, and YR2 follow-up. For work satisfaction, only participants that reported being employed were included in the analysis. Hence, participants who stated that they were retired or indicated that they were undertaking educational studies were excluded from work satisfaction analyses. However, as those patients met the inclusion criteria for the study, they were included in the HUI-3 and SSQ analyses.

### 3.2. Socio-Demographics

The mean age of participants was 46.7 (SD ± 12.6) years of age, and about two-thirds were female (63%). Participants were located in six different countries with most residing in South Africa (46.5%) and Spain (31.5%). Nine out of ten received one CI. The etiology of participants’ HL displayed that it was unknown what had caused the HL in approximately half of the cases. More than half of the participants reported to have tinnitus pre-implantation. Seventy percent of participants stated to be employed, and the majority were in full-time employment. Of the employed participants, the largest proportion were academic professionals (32.6%) followed by semi-skilled trained workers (25.8%), non-academic professionals (23.6%), and unskilled workers (10.1%). A smaller group of participants reported to have “other” occupations (7.9%). Only nine participants of the sample stated that they had a comorbid condition. A more detailed account of participants’ socio-demographic and clinical background information at baseline can be seen in Table 1.

### 3.3. Health-Related Quality of Life

Results for HUI-3 scores between baseline, YR1, and YR2 follow-up showed significant changes in HRQoL values for the attributes hearing, speech, emotion, cognition, and the overall health state utility score (Table 2). For the attributes, a significant change could be seen for hearing, which showed a change in score of 0.087 between baseline and YR1 and a further significant increase of 0.017 between YR1 and YR2 (*p* = 0.020). The aggregated difference between baseline and YR2 for hearing was 0.104 (*p* < 0.001). For speech, there was a significant change in scores of 0.015 between baseline and YR1 and YR2 follow-ups (*p* < 0.010). The attribute emotion had a significant difference of 0.0047 (*p* < 0.001) between baseline and YR1 and YR2. Cognition showed a significant change in scores of −0.006 (*p* < 0.004) between baseline and YR1 and YR2 follow-ups. For the health utility score, a significant increase of 0.169 (*p* < 0.001) was seen between baseline and YR1 and YR2 follow-up.

### 3.4. Hearing Disability

The result for hearing disability measured by the SSQ instrument showed that there was an improvement in scores for patients post-CI implantation between baseline, YR1, and YR2 follow-ups (Table 3). Changes in SSQ scores across all three domains and for the global score were significant between baseline and across YR1 and YR2 (*p* < 0.01, but no domain was significant between YR1 and YR2).

**Figure 1:**
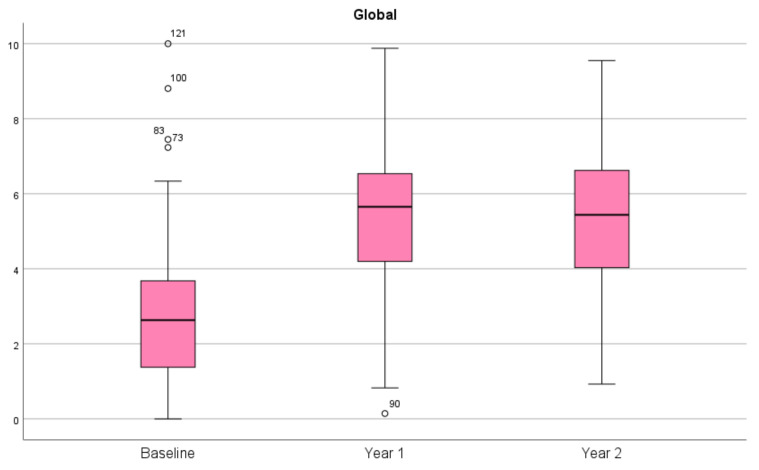
Boxplots for the SSQ global scores at baseline, year 1 and year 2 follow-up.

A visual display of the distribution of the data from the aggregated SSQ global score at baseline, YR1, and YR2 follow-up is shown as boxplots in Figure 1. As can be seen in the boxplots, the median slightly shifts across the different time points and fewer outliers are present at the YR1 and YR2 follow-ups. The boxplots also visually reflect the increased mean scoring values of the global score between baseline, YR1, and YR2 follow-up across all three SSQ domains.

### 3.5. Work Satisfaction

Eighty-nine patients in the sample were employed at baseline, as some individuals in the sample recently retired or were currently undertaking studies (Table 4). The employment number slightly decreased to 86 YR1 and to 83 employed participants at the YR2 follow-up due to participants retiring or commencing studies. The majority of the employed participants stated that they were satisfied with their work at baseline, which increased significantly post-CI implantation across the YR1 and YR2 follow-ups (*p* = 0.017). A significant increase could, however, not be seen between YR1 and YR2. Patients also stated that they, to a lesser extent, experienced their hearing ability to have a negative impact on the satisfaction of executing their daily work at YR1 and YR2 post-CI as compared to baseline. At baseline, patients’ summary score for their mean impact of hearing on their work was 2.521 (SD ± 0.852), indicating that, on average, their hearing negatively impacted their ability to work sometimes/most of the time. At YR1, patients scored their mean impact of hearing on their work at 1.603 (SD ± 0.640), indicating that, on average, their hearing impairment negatively impacted their ability to work not at all/sometimes. At the YR2 follow-up, the mean summary score was estimated at 1.575 (±0.622). Hence, there was a significant difference in mean score between baseline and across the YR1 and YR2 follow-ups of 0.946 (*p* < 0.001)

## 4. Discussion

This study identified overall increases in HRQoL that were significant for the attributes hearing, speech, emotion, and the aggregated health utility value between baseline and across the follow-ups in individuals who received CI implants. However, there were more prominent increases in HRQoL between baseline and YR1 and YR2 follow-ups compared to between YR1 and YR2. This suggests that there may be a larger initial effect post-CI implantation that levels out and potentially remains steady over time. A possible explanation that aligns with this finding may be that respondents attained stable CI programming parameters after the first switch-on, meaning stimulation levels usually obtain a fast evolution during the first weeks after activation of the sound processor. Then, the evolution becomes slower, and the programming parameters tend to be stable about six months after the initial switch-on [22]. The stable CI fitting parameters could also potentially explain the findings for the SSQ scores which presented a prominent increase in scores between baseline and YR1 and YR2 before stabilising between YR1 and YR2, except for speech, which showed a significant change between YR1 and YR2 follow-ups. There were smaller declines in HRQoL scoring for the attributes emotion, cognition, and health utility during the time of follow-up; those changes were not significant and are not necessarily related to hearing function, as the majority of SSQ domains and the global score showed overall increases. The health utility score in this current study reported 0.481 at baseline, and 0.653 and 0.650, at YR1 and YR2, respectively, which also shows similarity to the findings of a previous IROS study [23]. The previous study included individuals aged 18–80 years of age, had a smaller sample size (*n* = 70), and presented a health utility score of 0.49 at baseline and 0.56 at follow-up [23]. The substantially lower health utility reported in that study at YR1 post-CI in comparison to the current may be because they included older age participants, which likely had more comorbidities, hence, negatively impacting the possibility to increase the health utility further post-CI.

Comparing the baseline health utility score for individuals with HL in this study, which was estimated at 0.481, with other chronic diseases from a Canadian study also shows that the mean health status is worse than for the utility norms of living with the effects of stroke (0.581), urinary incontinence (0.621), and mood disorders (0.643). Furthermore, the improved scoring at YR1 and Y2, set at 0.653 and 0.650, respectively, is similar to the utility norms for chronic obstructive pulmonary disease (COPD) (0.649) [24]. This indicates that the effects of HL on HRQoL and daily living may be considered worse than for many other severe chronic illnesses. Moreover, even though individuals get Cis, they are still considered to be at the very same level as, for example, debilitating conditions such as COPD. Nonetheless, as this study establishes that the health utility score significantly increased by 0.169 (*p* < 0.001) between baseline and YR1 and YR2 follow-up, this denotes considerable clinical importance of CIs, as differences of 0.03 are considered clinically meaningful [19]. Similarly, for the attribute hearing as a single attribute, score differences of 0.05 are considered clinically important for the HUI-3 instrument [19], and it was shown that the score for hearing increased by 0.087 between the baseline and YR1 (*p* = 0.020) and 0.104 (*p* < 0.001) between the baseline and YR2, which suggests a substantial and clinically important improvement.

The findings for work satisfaction revealed that the majority of the employed participants were generally satisfied with their work at baseline (86.5%), a number that increased at YR1 follow-up (96.5%) and had a smaller drop in YR2 (91.6%). Participants also reported that, to a lesser extent, their HL negatively impacted their ability to work between the baseline and YR1 and YR2 follow-ups. This result aligns with a previous systematic review concluding that receiving CIs had a positive effect on the working life of individuals with HL in terms of better work performances related to, e.g., improved communication with colleagues and increased functioning [15]. From a health-economic perspective, it has been reported in a cost-benefit study that working-aged adults with profound HL have presented positive net benefits for CIs implanted at any age during working life [25]. Audiologists may not routinely ask nor have the time to enquire about to what extent individuals with HL are satisfied with their work situation, as the focus of discussion often centers around aural rehabilitation using hearing aids or CIs [26]. Nonetheless, data on work satisfaction may be important, as HL impacts individuals on social, physical, biological, medical/clinical, psychosocial, and normative levels [1,4]. Thus, this information can be used by decision makers and is relevant for public policy, as it indicates that CIs, in addition to increased HRQoL, hearing quality, and higher work satisfaction, may promote a higher productivity gain due to better work performance.

Limitations of the study include that there was no control group, which weakened the ability to draw conclusions about outcomes due to threats associated with validity. However, being a pre- and post-study with two follow-ups gives the advantage of enabling the assessment of patterns that may occur over a longer period that could not be observed over shorter periods of time. A further strength of this study is that the reliability was controlled for by using the instruments HUI-3 and SSQ to assess HRQoL. These instruments have been validated for responsiveness for individuals with HL and used in numerous studies for this patient group [20,27]. In addition, the generic HRQoL instrument HUI-3 also includes hearing as an attribute, which is not seen in most other generic instrumentation. However, the use of the non-validated work satisfaction questionnaire is a limitation of the study. It should also be noted that other personal and work-related factors that go beyond hearing loss may have contributed to participants’ perceptions of their work satisfaction. Another limitation of the study is that the studied population only included a sub-sample of working-age adults (18–65 years of age), hence, a limitation since adults older than 65 years of age in the workforce were not included. A methodological limitation in regard to HRQoL was that very limited data were available on comorbidities, as this was not captured for the majority of patients. Comorbidities are associated with decreases in health status and are considered to be an important aspect when interpreting HRQoL results [28]. Another limitation related to the work satisfaction outcome was that the instrumentation used to collect these data provided limited information on the extent to which the individual relied on or needed hearing in their work setting. This would have been relevant information as the need for functional hearing varies in relation to the work tasks performed and the workplace. Thus, CIs may impact the degree of work satisfaction depending on whether work tasks rely on communication and hearing ability. Social desirability bias and gender bias may also have impacted outcomes since aggregate data have shown that women typically report higher job satisfaction [29]. Another aspect that may have influenced the results is that there may be important cultural differences across the included countries that might have played a role in the outcomes. Finally, the rehabilitation trajectory following implantation can differ between clinics, which may lead to differences in patient outcomes between countries.

## 5. Conclusions

In conclusion, overall hearing quality increased post-CI and work satisfaction improved. With the increasing prevalence of hearing disabilities, this is pertinent knowledge that supports the use of CIs for hearing rehabilitation, which may promote better HRQoL and work satisfaction.

## Figures and Tables

**Table 1:** Participants’ socio-demographic and clinical background data at baseline, *n* = 127.

Participant Characteristics, (*n* = 127)	*n* (%)
Age (years), mean ± SD (min–max) *	46.7 ± 12.6 (18.7–65.2)
Gender	
Female	80 (63.0)
Male	47 (37.0)
Country of residence	
Colombia	2 (1.6)
Germany	14 (11.0)
Hungary	1 (0.8)
Poland	11 (8.7)
South Africa	59 (46.5)
Spain	40 (31.5)
Number of CI devices implanted	
1	120 (94.5)
2	7 (5.5)
Etiology of hearing loss	
Acoustic neuroma	1 (0.8)
Chronic Otitis	2 (1.6)
Congenital Atresia	4 (3.1)
Hereditary	12 (9.4)
Measles	7 (5.5)
Meniere’s disease	3 (2.4)
Meningitis	10 (7.9)
Noise exposure	5 (3.9)
Otosclerosis	12 (9.4)
Ototoxic drugs	11 (8.7)
Unknown	60 (47.2)
Tinnitus	
Presence of tinnitus pre-implant	82 (64.6)
No presence of tinnitus pre-implant	42 (33.1)
Unknown if tinnitus was present pre-implant	3 (2.4)
Employment status	
Employed	89 (70.1)
Unemployed	38 (29.9)
Extent of employment **	
Part-time	15 (16.9)
Full-time	74 (83.1)
Type of occupation **	
Unskilled	9 (10.1)
Semi-skilled (training)	23 (25.8)
Professional (non-academic)	21 (23.6)
Professional (academic)	29 (32.6)
Other	7 (7.9)
Comorbidities	
Yes	9 (7.1)
No	118 (92.9)

* Missing data for 5 participants, *n* = 122; ** Only employed individuals included *n* = 89.

**Table 2:** HUI-3 attributes and utility scores for patients at baseline and YR1 and YR2 follow-up, *n* = 127.

(Mean ± SD)	Baseline	YR1	YR2	*p*-Value ^a^ YR1–YR2	*p*-Value ^b^ Baseline—YR1/YR2
Vision	0.981 (±0.038)	0.982 (±0.034)	0.980 (±0.031)	0.599	0.562
Hearing	0.763 (±0.125)	0.850 (±0.093)	0.867 (±0.075)	0.020	<0.001
Speech	0.966 (±0.055)	0.980 (±0.043)	0.981 (±0.045)	0.848	0.010
Emotion	0.915 (±0.141)	0.967 (±0.071)	0.962 (±0.077)	0.464	<0.001
Pain	0.958 (±0.074)	0.965 (±0.075)	0.967 (±0.057)	0.979	0.613
Ambulation	0.987 (±0.052)	0.988 (±0.041)	0.989 (±0.042)	0.822	0.814
Dexterity	0.995 (±0.034)	0.995 (±0.019)	0.996 (±0.020)	0.527	0.405
Cognition	0.969 (±0.072)	0.969 (±0.077)	0.963 (±0.070)	0.197	0.004
Utility score	0.481 (±0.265)	0.653 (±0.248)	0.650 (±0.200)	0.377	<0.001

^a^ Wilcoxon Signed rank test; ^b^ Friedman’s test. YR1 = Year 1 follow-up. YR2 = Year 2 follow-up.

**Table 3:** Average disability of hearing domain and global scores at baseline and YR1 and YR2 follow-up, *n* = 127.

(Mean ± SD)	Baseline	YR1	YR2	*p*-Value ^a^ YR1–YR2	*p*-Value ^b^ Baseline–YR1/YR2
Speech	2.35 (±1.84)	5.13 (±2.02)	5.25 (±2.10)	0.059	<0.01
Spatial	2.47 (±1.98)	4.93 (±2.15)	4.84 (±2.17)	0.857	<0.01
Qualities	3.28 (±2.08)	5.90 (±1.88)	5.94 (±1.93)	0.401	<0.01
Global	2.73 (±1.80)	5.35 (±1.86)	5.37 (±1.90)	0.172	<0.01

^a^ Wilcoxon Signed rank test; ^b^ Friedman’s test. YR1 = Year 1 follow-up. YR2 = Year 2 follow-up.

**Table 4:** Employment and work satisfaction across baseline and YR1 and YR2 follow-up, *n* = 89.

	Baseline, *n* = 89 *n* (%)	YR1, *n* = 86 *n* (%)	YR2, *n* = 83 *n* (%)	*p*-Value YR1–YR2	*p*-Value Baseline—YR1/YR2
Employed	89 (70.1)	86 (67.7)	83 (65.4)	0.607 ^a^	0.340 ^b^
Full time	74 (83.2)	70 (81.4)	68 (81.9)		
Part-time	15 (16.8)	16 (18.6)	15 (18.1)	0.625 ^a^	0.417 ^b^
Satisfied with work					
Yes	77 (86.5)	83 (96.5)	76 (91.6)		
No	12 (13.5)	3 (3.5)	7 (8.4)	0.125 ^a^	0.017 ^b^
Hearing ability affects work satisfaction negatively					
Not at all	9 (10.1)	42 (33.1)	39 (30.7) *		
Sometimes	40 (44.9)	40 (31.5)	40 (31.5) *		
Most of the time	25 (28.1)	3 (2.4)	2 (1.6) *		
Always	15 (16.9)	1 (0.8)	1 (0.8) *		
Summary score (±SD) ^†^ **	2.521 (±0.852)	1.603 (±0.640)	1.575 (±0.622)	0.736 ^c^	<0.001 ^d^
Has your CI/s impacted your daily work over the last 12 months?					
Yes	N/A	79 (91.9)	72 (86.7)	0.302 ^a^	
No	N/A	7 (8.1)	11 (13.3)		

^†^ Hearing ability affecting work satisfaction negatively converted to a mean score; * Missing one value, *n* = 82; ** Missing values, *n* = 73; ^a^ McNemar’s test; ^b^ Cochran’s Q test; ^c^ Wilcoxon ranked test; ^d^ Friedman’s test. YR1 = Year 1 follow-up. YR2 = Year 2 follow-up.

## Data Availability

Aggregated, anonymized study data will be made available upon reasonable request by contacting the corresponding author.
